# A New Approach for Prostate Cancer Diagnosis by miRNA Profiling of Prostate-Derived Plasma Small Extracellular Vesicles

**DOI:** 10.3390/cells10092372

**Published:** 2021-09-09

**Authors:** Lidia Zabegina, Inga Nazarova, Nadezhda Nikiforova, Maria Slyusarenko, Elena Sidina, Margarita Knyazeva, Evgenia Tsyrlina, Sergey Novikov, Sergey Reva, Anastasia Malek

**Affiliations:** 1 Department of Radiotherapy, Subcellular Technology Laboratory, N.N. Petrov National Medical Research Center of Oncology, 197758 St. Petersburg, Russia; oblaka12@mail.ru (I.N.); niki2naden_ka@mail.ru (N.N.); slusarenko_masha@mail.ru (M.S.); sidina@mail.ru (E.S.); margo9793@gmail.com (M.K.); evg.tsyrlina@gmail.com (E.T.); krokon@mail.ru (S.N.); 2 Oncosystem Ltd., 121205 Moscow, Russia; 3 Department of Andrology and Urology, Research Center of Urology, Pavlov First Saint Petersburg State Medical University, 197022 St. Petersburg, Russia; sgreva79@mail.ru

**Keywords:** prostate cancer, diagnostics, small extracellular vesicles, SEVs, microRNA, miRNA, PSMA, PSMA(+)SEVs, click chemistry

## Abstract

Vesicular miRNA has emerged as a promising marker for various types of cancer, including prostate cancer (PC). In the advanced stage of PC, the cancer-cell-derived small extracellular vesicles (SEVs) may constitute a significant portion of circulating vesicles and may mediate a detectable change in the plasma vesicular miRNA profile. However, SEVs secreted by small tumor in the prostate gland constitute a tiny fraction of circulating vesicles and cause undetectable miRNA pattern changes. Thus, the isolation and miRNA profiling of a specific prostate-derived fraction of SEVs can improve the diagnostic potency of the methods based on vesicular miRNA analysis. Prostate-specific membrane antigen (PSMA) was selected as a marker of prostate-derived SEVs. Super-paramagnetic beads (SPMBs) were functionalized by PSMA-binding DNA aptamer (PSMA–Apt) via a click reaction. The efficacy of SPMB–PSMA–Apt complex formation and PSMA(+)SEVs capture were assayed by flow cytometry. miRNA was isolated from the total population of SEVs and PSMA(+)SEVs of PC patients (n = 55) and healthy donors (n = 30). Four PC-related miRNAs (miR-145, miR-451a, miR-143, and miR-221) were assayed by RT-PCR. The click chemistry allowed fixing DNA aptamers onto the surface of SPMB with an efficacy of up to 89.9%. The developed method more effectively isolates PSMA(+)SEVs than relevant antibody-based technology. The analysis of PC-related miRNA in the fraction of PSMA(+)SEVs was more sensitive and revealed distinct diagnostic potency (AUC: miR-145, 0.76; miR-221, 0.7; miR-451a, 0.65; and miR-141, 0.64) than analysis of the total SEV population. Thus, isolation of prostate-specific SEVs followed by analysis of vesicular miRNA might be a promising PC diagnosis method.

## 1. Introduction

Prostate cancer (PC) is one of the most common cancers among men. PC mortality rate is strongly correlated to the stage of the disease when first diagnosed. According to the St. Petersburg population-based cancer registry, the five-year relative survival rate of PC patients drops dramatically with increasing disease stage: 100% (stage I), 100% (stage II), 69% (stage III), and only 24% (stage IV) [1]. Data arising from other sources including the United States Surveillance, Epidemiology, and End Results (SEER) database demonstrate a similar tendency. This indicates an urgent need to develop a new, effective approach to detect PC earlier and increase the proportion of patients with a good prognosis. Current diagnostics of PC include serum prostate-specific antigen (PSA) determination, digital rectal examination (DRE), transrectal ultrasound (US), and US-guided tissue biopsy [2]. PSA and related markers, such as (−2)proPSA, and the prostate health index (PHI) have become widely used; however, their wide application did not considerably improve PC diagnostics, mostly due to low specificity. The effectiveness of DRE and US depends on the experience of the physician [3] and still lack appropriate standardization [4]. Tissue biopsy is an expensive and invasive procedure. Thus, the discovery of easily testable and highly specific markers of PC would fill the gap between PSA testing and tissue biopsy and would assist with the management of patients with increased PSA levels to improve the overall effectiveness of PC diagnostics.

Circulating with plasma, small extracellular nanovesicles (SEVs), especially exosomes, are recognized as a promising marker of different cancers [5], including PC [6]. The involvement of SEVs in PC development and dissemination was comprehensively summarized in recent reviews [7,8]. Different components of plasma SEVs, including exosomal RNA and proteins, have been studied as PC markers in dozens of studies (listed in [8]). Vesicular microRNAs (miRNAs) are the markers of PC most extensively studied over the past few years [9]. However, the results of these investigations are still not sufficiently reproducible, most likely due to methodological particularities and the fundamental problem of the high heterogeneity of plasma SEVs. The population of plasma SEVs derives from different cells in the bodies in an as-yet unknown ratio. As a small tumor of prostate gland does not alter considerably the composition of the plasma SEV population, the quantification of prostate-derived vesicles with diagnostic purpose would be of little value. The isolation followed by qualitative analysis of the population of prostate-derived SEVs may present a more promising approach for vesicle-based liquid biopsy.

The mature prostate gland is composed of columnar epithelial cells lining the prostate lumen and elongated basal cells separating the lumen from the stroma. PC can arise from both types of cells. Maintaining a certain level of tissue differentiation, transformed epithelial cells of the prostate show a tissue-specific profile of membrane proteins and secrete SEVs bearing tissue-specific markers. For instance, zinc metalloenzyme glutamate carboxypeptidase II (GCPII) or prostate-specific membrane antigen (PSMA) is enriched in prostate epithelial cells and are detected in the membrane of the prostate and prostate-cancer-secreted SEVs [10,11]. We hypothesized that a population of PSMA(+)SEVs would much better reflect cancer-specific alterations in the miRNAs occurring in the cells of the prostate gland than the total population of plasma SEVs. To explore this hypothesis, we developed a method to isolate PSMA(+)SEVs using super-paramagnetic beads functionalized by PSMA-specific DNA aptamer [12] via the click reaction. In combination with the previously described two-phase polymer system for plasma SEV isolation [13], the proposed technique provides a scaffold for an effective and economic approach to vesicle-based liquid biopsy. We analyzed four PC-associated miRNAs in PSMA(+)SEVs and the total population of plasma vesicles, and compared the diagnostic potency of these tests. The obtained results confirmed that the isolation of PSMA(+)SEVs increases the sensitivity of PC-associated miRNAs analysis and improves the values of the most important diagnostic parameters.

## 2. Materials and Methods

### 2.1. Patients

Plasma samples were obtained from patients undergoing treatment at N.N. Petrov National Medical Research Centre of Oncology and Pavlov First Saint Petersburg State Medical University. The study included patients with a histologically confirmed diagnosis of prostate cancer (*n* = 55) who met the specified criteria: age 56–70 years (median age was 63 years); no chronic or metabolic diseases; prostate cancer stage, T1c–T2c N0 M0; and Gleason score, 5–7. Plasma from healthy male donors (*n* = 30) age 55–70 years (median age was 61 years) was obtained from the blood transfusion department of the N.N. Petrov National Medical Research Centre of Oncology. Donors and patients gave informed consent to participate in the study.

### 2.2. Plasma Sampling and Preparation

Blood samples (5 mL) were placed in EDTA tubes. Plasma was immediately separated by centrifugation for 15 min at 1500× *g* and then stored at −80 °C. Before analysis, the frozen plasma was slowly thawed at 4 °C, sequentially centrifuged at 300, 1500, and 2500× *g*, and filtered through a 0.2 µm syringe filter (Minisart High Flow, Sartorius, Goettingen, Germany) to remove cellular debris. Plasma prepared by this method is hereafter referred to as pellet pure plasma (PPP) and was used for all experiments.

### 2.3. Labeling and Isolation of the Total Population of Plasma Extracellular Nanovesicles (SEVs)

The PPP was stained with lipophilic dye and fractionated by size-exclusion chromatography (SEC) as recently described [14]. Briefly, 2 mL of PPP was mixed with 2 μL of Vybrant™ Dil Cell-Labeling Solution dye solution (50 μM in DMSO; Thermo Fisher Scientific, Walthman, MA, USA) incubated at 37 °C for 20 min under moderate stirring. Each sample of stained plasma was loaded into the SEC column (HansaBioMed, Tallin, Estonia), and 23 fractions of 500 µL were collected in according to the producer’s manual. Fractions 9–11 were mixed and used for further experiments.

### 2.4. Total EVs Isolation by Two-Phase Polymer System

The total EV population was isolated from PPP using a two-phase polymer system as previously described [13], with slight modifications. Briefly, a solution of dextran (450–650 kDa, 1.5%) and polyethylene glycol (35 kDa, 3.5%) (both from Sigma-Aldrich, St. Louis, MO, USA) was prepared in the required volume of PPP. To prepare a protein-depleting solution (PDS), the same amounts of polymers were dissolved in an equal volume of phosphate-buffered saline (PBS). The PPP-based solution was centrifuged for 10 min at 1000× *g* to achieve phase separation. The upper phase, containing plasma proteins, was replaced with PDS, then the solution was well-mixed and centrifuged again. The upper phase formed during the second round of centrifugation was removed. The lower phase containing SEVs was resuspended in 100 μL of PBS.

### 2.5. Nanoparticle Tracking Analysis (NTA)

The size and concentration of the isolated vesicles were measured using a NanoSight NS300 analyzer (Malvern Panalytical, Malvern, UK) at camera level: 10, shutter slider: 696, slider gain: 55, and threshold level: 5. Each sample was pumped through the observation camera to record 5 measurements for 30 s for 749 frames at different microvolumes of the same sample. Based on the results of five measurements, the average values of the size and concentration of the nanoparticles in the suspension were calculated. The data were processed in Nanosight NTA 3.4.

### 2.6. Transmission Cryo-Electron Microscopy (Cryo-TEM)

Visualization was performed using cryo-electron microscopy on a JEOL JEM-1400 transmission electron microscope (JEOL Ltd., Tokyo, Japan) at the Research Resource Center for Molecular and Cell Technologies of Saint-Petersburg State University. The SEV samples at a concentration of 1012 particles/mL were deposited on carbon-coated copper mesh/lacey carbon-supported copper grids, 50 nm in size (Sigma-Aldrich, St. Louis, MO, USA). The excess sample was removed with filter paper. After, the sample was immersed in liquid ethane for rapid freezing and transferred to a cryostat for subsequent analysis using a cryo-microscope.

### 2.7. Bead-Assisted Flow Cytometry of the Total SEV Population

Plasma SEV surface markers were detected by flow cytometry using an Exo-FACS kit (HansaBioMed, Tallinn, Estonia) according to the manufacturer’s protocol. SEVs were absorbed nonspecifically to the surface of latex microparticles. Tetraspanins CD9 and CD63 were labelled using antibodies conjugated with fluorescent labels PE (CD9-PE, 312105, BioLegends, San Diego, CA, USA) and FITC (CD63-FITC, Ab18235, Abcam, Cambridge, UK). Measurements were performed on a Cytoflex analyzer; data were processed using CytExpert software (both from Beckman Coulter, Brea, CA, USA).

### 2.8. Dot Blotting

SEVs were isolated from the PPP (2 mL) of healthy donors with a two-phase polymer system and dissolved in 100 µL of PBS. SEV samples (0.4 µL) were applied to a nitrocellulose membrane and allowed to dry. The membrane was incubated in blocking buffer containing 5% bovine serum albumin (BSA) in tris-buffered saline (TBS) at room temperature for 20 min under moderate stirring. Membranes with spotted samples were incubated with primary antibodies against PSMA (133579, Abcam, Cambridge, UK), CD9 (312102, BioLegend, San Diego, CA, USA), CD63 (353039, BioLegend, San Diego, CA USA), and HSP70 (kindly provided by the author of Patent RF, 2722398) at an equivalent concentration of 1 ng/mL in 0.2% BSA solution at 4 °C overnight. Unbound primary antibodies were washed with TBS. Membranes were incubated first in a blocking buffer (inactivated plasma centrifuged for 5 min at 17,000× *g* and filtered through a 0.1 µm syringe filter), then with secondary antibodies, either goat anti-mouse IgG (6789, Abcam, Cambridge, UK) or goat anti-rabbit IgG (7171, Abcam Cambridge, UK) at a concentration of 0.13 ng/mL in 0.1% BSA solution at 4 °C for 20 min. Finally, membranes were washed twice in TBS and evaluated with Pierce^TM^ ECL Western Blotting Substrate and the iBright^TM^ FL1500 Imaging System (both from ThermoFisher Scientific, Walthman, MA, USA).

### 2.9. Formation of Complexes for PSMA(+)SEVs Sorption

The complex was composed of super-paramagnetic beads (SPMBs) with the surface functionalized by –N_3_ groups (Click Chemistry Tools, Phoenix, AZ, USA) and a PSMA-specific DNA-aptamer (PSMA–Apt) modified by a dibenzocyclooctyne group (DBCO) at the −5′ end. The sequence of the PSMA-specific aptamer (DBCO-5′-GAA TTC GCG TTT TCG CTT TTG CGT TTT GGG TCA TCT GCT TAC GAT AGC AAT GCT-3′) was adopted from [12] and synthesized by Syntol Ltd., Moscow, Russia. Complex SPMB–PSMA–Apt was formed by the reaction of azide-alkyne cycloaddition. First, 1 µL of SPMB (1 mg/mL) was incubated in a 200 µL I-Block™ Protein-Based Blocking Reagent (T2015, ThermoFisher Scientific, Walthman, MA, USA) at 4 °C for 1 h to prevent nonspecific binding. The SPMBs were then washed twice in 200 µL of PBS and resuspended in 100 µL of PBS. Then 1 µL of PSMA–Apt solution (100 pM) was added to the suspension of blocked SPMB, mixed, and incubated for 3 h at room temperature. The SPMB–Apt complexes were washed twice with 200 µM of PBS to remove the unbound aptamers. To test the efficacy of the formed complexes, we used Cy5.5-labelled aptamer. In this case, the pellet of SPMB–Apt was resuspended in 100 µM of PBS and used for flow cytometry. To capture PSMA(+)SEVs, the pellet of SPMB functionalized by unlabeled aptamer was mixed with the suspension of SEVs.

### 2.10. PSMA(+)SEVs Sorption by the SPMB–Apt Complex

As described above, 100 µL of SEVs suspension was added to the pellet of SPMB-Apt complexes, mixed, incubated at 4 °C overnight under moderate stirring, washed twice in 200 µL of unbound SEVs, and resuspended in 100 μL of PBS. To assay the efficacy of PSMA(+)SEVs captured by flow cytometry, plasma SEVs were labeled and isolated by SEC as described in Section 2.3. To assay the efficacy of PSMA(+)SEVs captured by dot blotting, plasma SEVs were isolated with a two-phase polymer system, as described in Section 2.4.

### 2.11. Formation of Immuno-Beads for PSMA(+)SEVs Immune-Sorption

To isolate PSMA(+)SEVs, we used the complex of super-paramagnetic beads with the surface functionalized by streptavidin (SPMB-St, K0180, Sileks, Moscow, Russian Federation) and PSMA-specific antibodies (Ab-PSMA, PSMCC8, HyTest Ltd., Turku, Finland) modified by biotin. The complex of SPMB-St–PSMA-Ab was formed as previously described [15]. Briefly, the SPMB-St was washed 3 times in 200 µL of PBS. Then, 0.5 µL of PSMA-Ab (3.4 mg/mL) was mixed with 1 µL of SPMB-St (1 mg/mL) in 100 µL of PBS and incubated at 4 °C for 1 h. Then, unbound Ab was washed twice in 200 µL of PBS; SPMB-St–PSMA-Ab complexes were used in the following experiments.

### 2.12. MiRNA Isolation

RNA from the SEVs was isolated with an RNAGEM kit (MicroGem, Dunedin, New Zealand) according to the manufacturer’s protocol. Both the total population of SEVs and PSMA(+)SEVs were isolated from 2 mL of PPP. The total population of SEVs was isolated by a two-phase polymer system. PSMA(+)SEVs were isolated by SPMB–PSMA–Apt complexes. Proteolysis was performed in 50 µL of water solution containing 5 µL of 10× BLUE buffer and 0.2 µL of RNAGEM reagent at 75 °C for 5 min. The concentration of the RNA was estimated using a Qubit 1.0 fluorimeter.

### 2.13. RT-PCR

The isolated RNA was analyzed by reverse transcription (RT), followed by real-time polymerase chain reaction (PCR) using a commercial ALMIR kit: AL145-5p, AL221-3p, AL451a-3p, AL141-3p (Algimed Techno, Minsk, Belarus) according to the manufacturer’s protocol. RT-reaction mix contained 2 μL of total RNA solution (Section 2.11), RT primer (50 pM), M-MLV reverse transcriptase (40 U) and proprietary RT buffer in a final volume 10 μL. The RT reaction was conducted for 45 min at 25 °C, then for 5 min at 85 °C to inactivate the enzymatic activity of reverse transcriptase. The following PCR was performed in a reaction volume of 20 μL including: RT reaction mix (4 μL), forward and reverse primers (each for 250 pM), FAM-labelled probe (150 pM) and 10 μL of 2xPCR master mix. Conditions of PCR: 5 min—95 °C, then 40 cycles: 5 s—95 °C; 15 s—60 °C. Any reaction was conducted in technical duplicate.

### 2.14. Statistics

The experimental data were processed using ImageJ, SigmaPlot 12.0, GraphPad Prizm 8, OriginPro 9.1, and CFX Manager Software 3.1. Statistical differences between groups of samples were evaluated using the nonparametric Mann–Whitney test. ROC analysis was used to assess the diagnostic significance of the developed method.

## 3. Results

### 3.1. Characteristics of the Total Population of Plasma Nanovesicles

**Figure 1:**
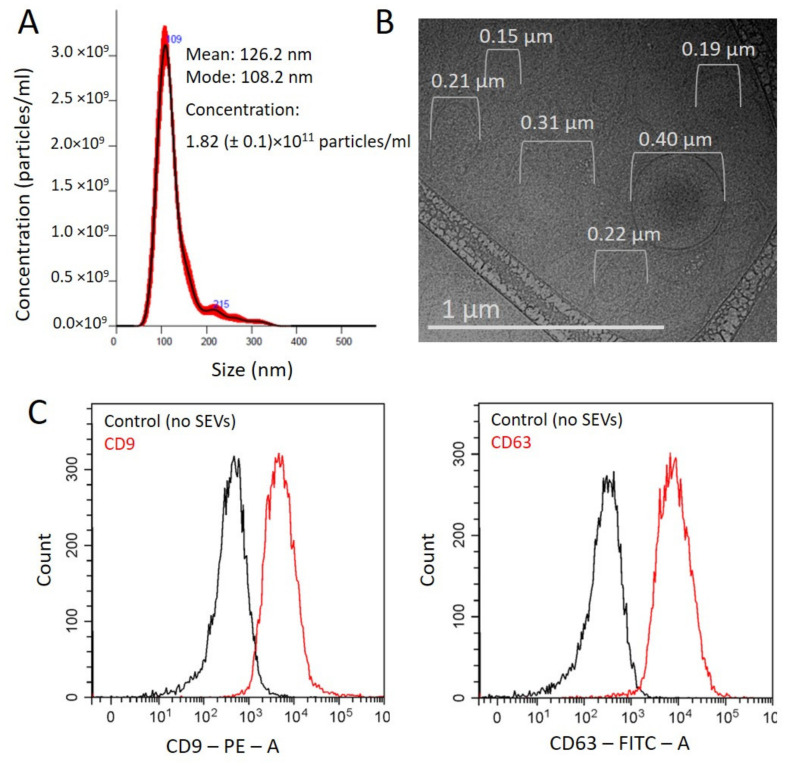
Characteristics of the total SEV population. (**A**) A representative example of NTA vesicles isolated by a two-phase polymer system. (**B**) Cryo-electron microscopy image of extracellular vesicles. (**C**) Detection of exosomal markers by flow cytometry. EVs attached to latex beads were stained with PC5.5-labelled antibodies to CD9 or FITC-labelled antibodies to CD63. Beads stained with antibodies were assayed as a negative control.

The total population of nanovesicles was isolated from pellet pure plasma (PPP) samples using a two-phase polymer system [13]. Unless otherwise specified, SEVs were isolated from 2 mL of PPP and resuspended in 100 μL of PBS. To estimate the size and concentration of the isolated vesicles by NTA, the suspension was again dissolved by PBS at 1:100. For each sample, results of five measurements were averaged during analysis. Concentration averaged for eight control samples used for validation of PSMA(+)SEV isolation method was 1.4 (±3) × 10^11^ particles/mL with averaged size 122 ± 13 nm. The concentration of the SEVs in individual samples used in further experiments was in a ranged from 1 × 10^11^ to 3 × 10^11^ particles/mL, with the predominant particle size ranging from 100 to 130 nm. A representative example is shown in Figure 1A. A cryo-TEM revealed that in the round form of isolated vesicles formed by a thin membrane, the average size of vesicles was 43 ± 16 nm (Figure 1B). Discrepancy of NTA and Cryo-TEM data was expectable due to methodological issues. Surface exosomal markers, tetraspanins CD9 and CD63, were detected by flow cytometry (Figure 1C). Given the size, shape, and presence of surface markers, we assumed that the isolated particles had a vesicular structure and were represented, at least in part, by exosomes.

### 3.2. Estimation of the Number of PSMA(+) Vesicles in the General Population

**Figure 2:**
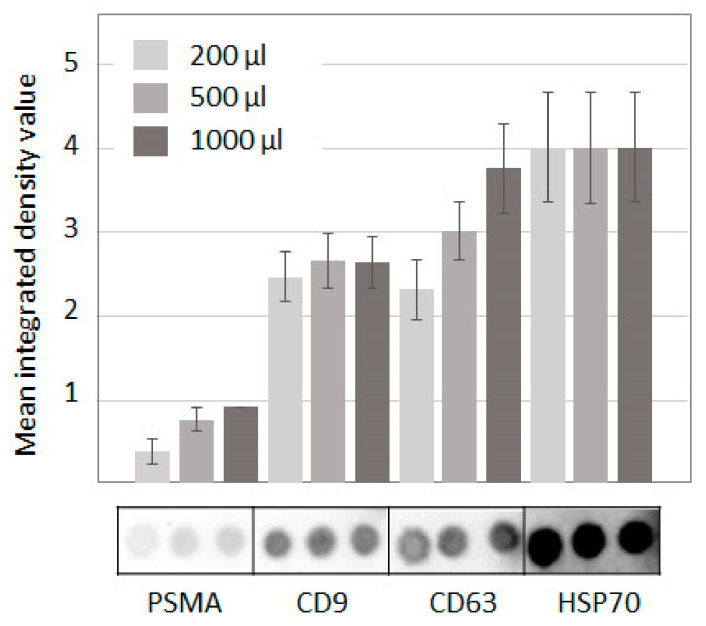
Membrane markers expression in plasma SEVs. Assessment of the relative concentration of membrane markers (PSMA, CD9, CD63, and HSP70) in the total population of plasma SEVs isolated from 200, 500, and 1000 μL of pellet pure plasma of healthy male donors by dot blotting. The integrated density values were obtained from spots, and the results of 8 participants were averaged.

Prostate-specific membrane antigen, PSMA (or folate hydrolase 1 (FOLH1)), a type 2 membrane glycoprotein, is expressed in various tissues [14], and is enhanced in the brain, intestine, and prostate [16]. PSMA overexpression is associated with PC development [17], with a high Gleason score of PC [18], whereas PSMA can be explored as a marker for the detection of recurrent disease and therapy with PSMA-targeted radioligands [19]. PSMA was detected in the membranes of SEVs secreted by PC cells in vitro [10], and the feasibility of PSMA(+)SEVs isolation from plasma was previously reported [11]. However, the relative amount of the PSMA(+)SEVs fraction in the plasma of healthy donors was never estimated. The relative mass of the prostate can be roughly assumed to be 0.03–0.05% of body weight (average body mass = 62 kg, average weight of a prostate = 20–30 g [20]). If we neglect the difference in the secretory activity and the degree of vascularization of all tissues, these percentages can be extrapolated to the relative amount of prostate-derived SEVs within the total population of plasma vesicles. This small amount can be difficult to detect. To evaluate relative amount of PSMA(+)SEVs, the total population of vesicles was isolated from PPP of healthy male donors (n = 8) age 55–65 (median age was 60 years), and expressions of exosomal surface markers (CD9, CD63, HSP70) and PSMA were measured in parallel by dot blotting. Since the limit of detection of PSMA(+)SEVs was unknown, each experiment was performed in triplicate using samples of SEVs isolated from 200, 500, and 1000 μL of PPP. The results are presented in Figure 2.

In contrast with our expectation, the amount of PSMA in plasma SEVs was comparable to amounts of other common markers. It accounted for 15–34% of CD9, 20–24% of CD63, and 9–22% of HSP70. Notably, the intensity of the signal from HSP70 was saturated in this experiment, whereas intensity of the signal from CD9 was close to its saturation level. If the intensity of CD63 and the PSMA expression in the vesicular membrane are comparable, the amount of PSMA(+)SEVs reached 20–24% from the total population of plasma CD63(+)SEVs. It seems unlikely that PSMA is so highly present in the membranes of prostate-derived SEVs to mediate such an intensive signal from a small number of vesicles. A more plausible explanation for the obtained results is the secretion of PSMA(+)SEVs by the cells of different tissues, not by cells of the prostate only. Thus, the fraction of PSMA(+)SEVs is apparently enriched by prostate-derived vesicles but does not consist of them exclusively; PSMA is not a very specific marker.

### 3.3. Formation and Evaluation of PSMA(+)SEV-Capturing Complex

**Figure 3:**
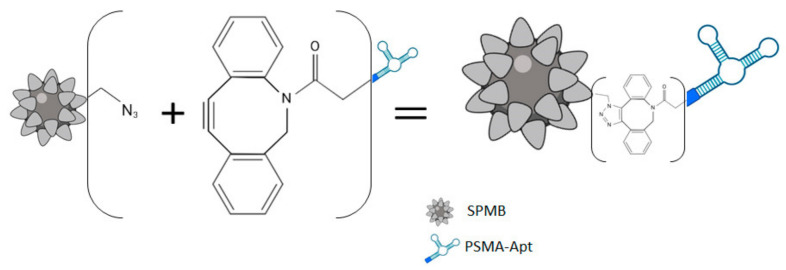
Scheme of SPMB–PSMA–Apt complex formation.

Next, we aimed to develop a new technology for the effective isolation of PSMA(+)SEVs by their capturing with a PSMA-specific DNA aptamer (PSMA–Apt) fixed to the surface of super-paramagnetic beads (SPMBs). We applied the click chemistry approach using SPMBs with an azide group and PSMA–Apt with dibenzenecyclooctine at the 5′ end (Figure 3).

**Figure 4:**
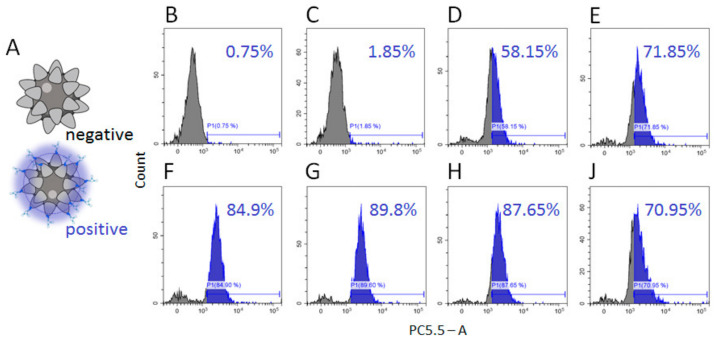
Efficacy of SPMB–PSMA–Apt formation. (**A**) Scheme of experiment: the signal from non-modified SPMBs was set as 0, SPMBs modified with Cy5.5-labeled aptamer were counted as positive events; (**B**–**G**) efficacy of complex formation with increased amounts of aptamer in a reaction mix (0.01, 0.1, 0.5, 0.7, 1, and 2 pM) for 3 h at room temperature; (**H**) efficacy of complex formation in the presence of DMSO (10%) in reaction mix (1 pM of Apt, 3 h, RT); (**J**) efficacy of complex formation in optimal conditions (1 pM of aptamer, 10% DMSO) overnight at a temperature of 4 °C.

To optimize the conditions of SPMB–PSMA–Apt complex formation, we used a DNA aptamer modified with cyanine 5.5 (Cy5.5) dye at the 3′ end, and assayed the efficacy of complex formation by flow cytometry. Selected results are presented in Figure 4.

We tested different conditions for complex formation including aptamer concentrations, the presence of dimethylsulfoxide (DMSO), temperature (RT vs. 4 °C), and reaction time (3 h vs. overnight). An increase in aptamer concentration in the reaction mix up to 2 pM was associated with an increase in the percentage of SPMB modified with Cy5.5. aptamer (Figure 4B–G); further increases in aptamer concentration did not improve the efficacy of complex formation. As a positive effect of DMSO in an analogic reaction was reported [21], we tested the efficacy of complex formation in the presence of 5%, 10%, and 20% DMSO in reactions with 1 and 2 pM of aptamers. We found that 10% DMSO led to the modification of 87.65% of SPMBs using an aptamer concentration of 1 pM (Figure 4H). We expected the increase in incubation time (overnight vs. of 3 h), or adjusting the reaction temperature (4 °C vs. RT) would improve reaction efficacy. However, the parameters did not increase the efficacy of SPMB–PSMA–Apt formation (Figure 4J). On the basis of obtained results, we set the optimal reaction conditions as: 1 µg SPMBs in 100 μL of PBS, 1 pM PSMA–Apt, 10% DMSO, RT, and 3 h of incubation time.

### 3.4. Efficacy of PSMA(+)SEVs Isolation

**Figure 5:**
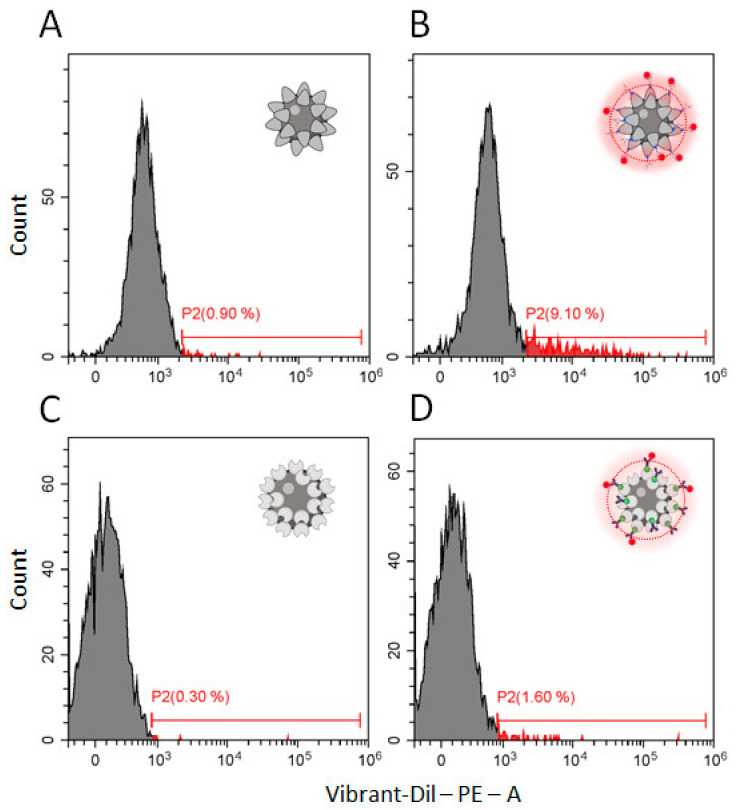
Efficiency of the PSMA(+)SEVs capture by complexes composed of PSMA-specific DNA aptamers and antibodies. (**A**) SPMBs with the surface functionalized by –N_3_ groups (no aptamer) incubated with Vibrant-Dil-labeled SEVs (negative control); (**B**) SPNBs modified with PSMA–Apt via alkyne-azide cycloaddition and incubated with Vibrant-Dil-labeled SEVs; (**C**) SPMBs with the surface functionalized by streptavidin (no antibodies) incubated with Vibrant-Dil-labeled SEVs (negative control); (**D**) SPMBs modified by antibodies against PSMA via a biotin–streptavidin link and incubated with Vibrant-Dil-labeled SEVs.

Next, we aimed to compare the efficacy of PSMA(+)SEVs capturing with complexes formed by either PSMA–Apt or antibodies against PSMA (PSMA-Ab). To form SPMB–PSMA–Apt complexes, we used PSMA–Apt without modification by Cy5.5. The formation of immuno-beads from SPMBs modified by streptavidin and biotin-labeled antibodies was reported previously [15]; herein, we used the same technology with validated antibodies against PSMA. An equal amount of SPMB (1 µg) with a diameter of 1 µm modified either by an –N_3_ or by a streptavidin group was functionalized either by PSMA–Apt or by PSMA-Ab as described in Section 2.9 and Section 2.11. Both complexes were incubated with equal amounts of SEVs stained with lipophilic dye (Vibrant Dil) and isolated from plasma of healthy male donors (n = 8) by SEC (Section 2.3 and [14]). The efficacy of PSMA(+)SEVs capture was estimated by flow cytometry; a representative example of one sample’s analysis is presented in Figure 5.

The SPMB non-functionalized by SPMA-binding ligands was used as a negative control: a certain level of nonspecific binding of Vibrant-Dil-labeled vesicles was detected in both cases (Figure 5A,C). In the group of eight samples, the percentage of positive events varied between 0.5% and 1.3% in the case of SPMBs functionalized by an –N_3_ group and between 0.2% and 0.7% in the case of SPMBs functionalized by streptavidin. These levels of nonspecific interaction were assumed to be acceptable. PSMA(+)SEVs were captured from the total population of plasma vesicles by SPMB–PSMA–Apt complexes with an efficacy of 6.5–10.8%, and by SPMB–SPMA–antibody complexes with an efficacy of 1.1–3.4% (Figure 5B,D). Comparing the efficacy of PSMA(+)SEVs isolation of the two methods from the same sample, the efficacy of SPMB–PSMA–Apt complexes was 4–6-fold higher than that of SPMA–SPMA–antibody complexes.

**Figure 6:**
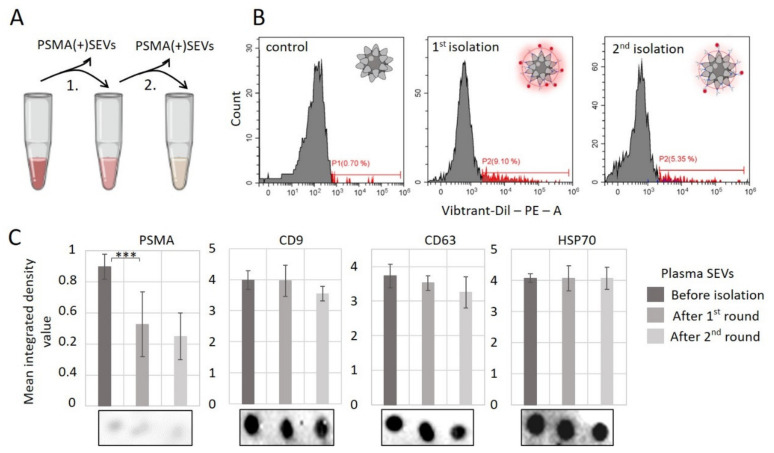
Evaluation of the efficiency of the isolation of PSMA(+)SEVs from the total population of plasma SEVs. (**A**) Experiment scheme. (**B**) Effectiveness of two consequent rounds of SPMA(+)SEVs capture was assayed by flow cytometry. SPMB non-functionalized by PSMA–Apt was stained with SEVs and assayed as a negative control. (**C**) The effectiveness of PSMA(+)SEVs depletion from the total population of plasma SEVs through two rounds of incubation with PSMB–PSMA–Apt complexes was analyzed by dot blotting. The amounts of PSMA, CD9, CD63, and HSP70 were assayed in parallel. Representative images from one sample’s analysis are presented. All images were digitalized by ImageJ, the results were averaged within the groups, the statistical significance of the difference between paired groups was assayed using the Mann–Whitney test: *** *p* < 0.0005.

As the amount of PSMA(+)SEVs was assessed as being significant, we wanted to evaluate the capabilities of the developed method and to test the effectiveness of PSMA(+)SEVs depletion from the total population of plasma vesicles. To prepare SEVs, samples of PPP were stained with Vibrant-Dil and fractionated by SEC as in the previous experiment. The PSMA(+)SEVs were captured from the general population during overnight incubation. The complexes were isolated, washed, and analyzed by flow cytometry, and the supernatant with unbound SEVs was used for the second round of PSMA(+)SEVs isolation with fresh complexes. After the second round of overnight incubation, complexes were isolated, washed, and analyzed by flow cytometry as well. In parallel, we analyzed the level of membrane vesicular markers (PSMA, CD9, CD63, and HSP70) in the reaction mix before the first incubation, after the first incubation, and after the second incubation with SPMB–PSMA–Apt complexes. The scheme of the experiment is presented in Figure 6A.

We used eight samples of SEVs isolated from the plasma of healthy male donors. SPMBs functionalized by an –N_3_ group (without PSMA–Apt) were stained overnight and assayed as the negative control (signal varied from 0.2% to 0.9%). Analysis of SPMB–PSMA–Apt complexes incubated with SEVs and washed revealed 6.7% to 10.3% positive events. The second round of SPMA(+)SEVs isolation with fresh complexes produced a much lower percentage of positive events (3.4–6.1%). Representative results of the experiment are presented in Figure 6B. In parallel, dot blotting revealed a significant decrease in PSMA in the reaction mixture; the reduction in CD9 and CD63 was negligible and statistically not significant; the amount of HSP70 did not change through the two rounds of PSMA(+)SEVs isolation (Figure 6C). Moreover, the first round of isolation was much more effective and resulted in the substantial depletion of PSMA(+)SEVs compared to the second round. Thus, the obtained results demonstrated the possibility of the successful isolation of PSMA(+)SEVs from the total population of vesicles from healthy donors plasma, so we suggested that using SPMB–PSMA–Apt complexes should also be effective with vesicles from PC patients.

### 3.5. Evaluation of the Diagnostic Potency of miRNAs from PSMA(+)SEVs

After the PSMA(+)SEVs isolation method was established, we aimed to test if the analysis of PC-related miRNAs from this specific population of SEVs has higher diagnostic potency than miRNA from the total population of plasma SEVs. We used material from healthy donors (n = 30) and patients with histologically confirmed PC (n = 55). miRNA was isolated from the general population and from the PSMA(+)SEVs in parallel. We selected four miRNAs involved in PC development: mir-141 [22], mir-145 [22], mir-221 [23], and mir-451a [24]. Mir-141 is reported to be consistently up-regulated in PC compared with healthy controls and has been suggested as a biomarker for biochemical failure and clinical outcome [25]. MiR-145 regulates tumor growth, invasion, and metastasis, and it also has a role in tumor angiogenesis and tumor stem cell proliferation [26]. Mir-221 is a cancer-associated miRNA, and its overexpression contributes to the progression of prostate carcinoma. The levels of miR-221 were significantly higher in aggressive prostate cancer tissue samples than in non-aggressive prostate cancer tissue samples [27]. MiRNA-451 influences the progression of tumorigenesis and drug resistance [28]. It was shown that miR-451 levels were significantly higher in exosomes from human prostate cancer cells under hypoxic conditions than in those under normoxic conditions [24].

**Figure 7:**
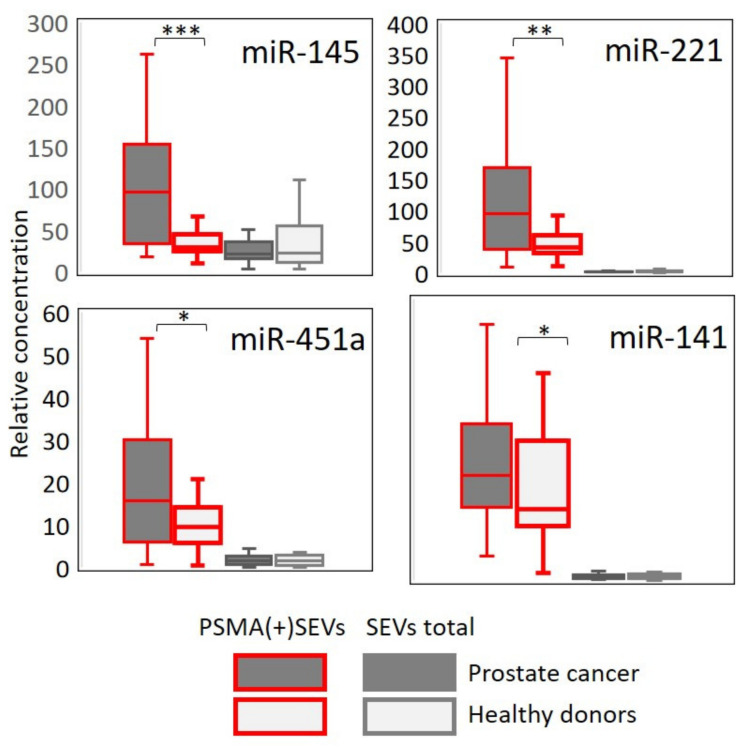
Comparative analysis of mir-145, mir-221, mir-451a, and mir-141 in plasma SEVs of prostate cancer patients and healthy donors. Plasma samples from PC patients’ (n = 55) and healthy donors’ (n = 30) RNA were used. From each sample, the total population plasma SEVs and PSMA(+)SEVs were assayed. RNA was isolated and RT-PCR analysis of miR-145, miR-221, miR-451a, miR-141, and miR-93 was performed in duplicate. Results were normalized versus miR-93 and averaged within groups. Statistical significance between paired groups (PC vs. healthy donors) was determined using the Mann–Whitney test: * *p* < 0.05, ** *p* < 0.005, and *** *p* < 0.00005.

All these molecules were expected to be detectable in the plasma SEVs of healthy donors according to our previous experiments and to the open database ExoCarta [29]. Semi-quantitative assessment of the concentration of the selected molecules was conducted by RT-PCR. The reactions were performed in duplicate, and the Ct values were averaged. Hsa-mir-93 was assayed in parallel and used as a reference to normalize the obtained data by the standard method: ΔCt = 2 (Ct^mir93^ − Ct^mirX^). Figure 7 shows the normalized values of the concentrations of the four selected miRNAs in two clinical groups (PC patients and healthy donors). Analysis was performed in parallel with RNA isolated from the total population of plasma SEVs (grey border) and from PSMA(+)SEVs (red border).

Analysis of miRNAs in the total population of plasma SEVs revealed a certain amount of these molecules; however, no difference between PC patients and healthy donors was detected. When RNA was isolated from the population of PSMA(+)SEVs, PC-associated miRNAs were confidently detected. The difference between groups of PC patients and healthy donors was statistically significant in all four cases.

**Figure 8:**
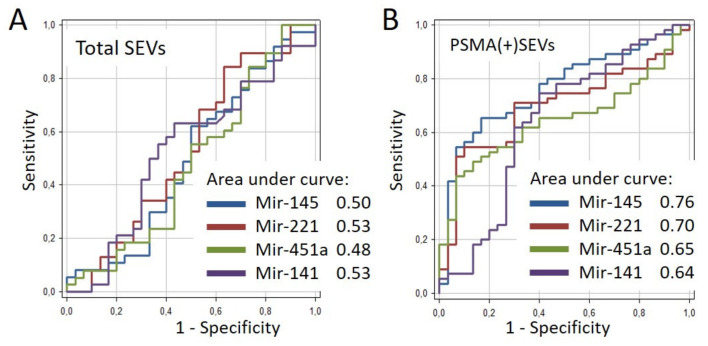
Evaluation of the diagnostic potential of the method. Receiver operating characteristic (ROC) curves for mir-145, mir-221, mir-451a, and mir-141 for total (**A**) and PSMA(+) (**B**) SEVs were created using the results from 85 samples: PC patients (n = 55) and healthy donors (n = 30) using Graph Pad Prism software.

Receiver operating characteristic (ROC) analysis was used to assess the diagnostic potential of miRNA from the total population of plasma SEVs and PSMA(+)SEVs. The results are shown in Figure 8. For the miRNAs isolated from the general population of SEVs, the area under the curve (AUC) value deviated slightly from the midline, which indicates the low predictive potential of the model. AUC values in the range from 0.64 to 0.76 were obtained for miRNAs isolated from the PSMA(+)SEVs, which indicates a good prognostic potential. The obtained results correlate with the data presented in Figure 8: the maximum difference between the groups of patients and healthy donors (*p* < 0.0005) and the highest AUC (0.76) value were observed for miR-145. A less prominent difference between clinical groups (*p* < 0.05) and lower AUC values (0.65 and 0.64) were observed for miR-451a and miR-141.

For a more detailed assessment of the diagnostic potential of miRNAs analysis from PSMA(+)SEVs, additional indicators of diagnostic significance were calculated (Table 1). However, these parameters depend on a cutoff level selected for calculation. Different ratios of the sensitivity and specificity, and positive and negative predictive values can be obtained by altering the cutoff levels. For instance, the combination of sensitivity, specificity, and accuracy for miR-145 varied from 93.3%, 53.6%, and 68.2% to 65.5%, 83.3%, and 71.8%, respectively. Thus, the diagnostic potential of the method can be adjusted to more effectively solve clinical problems: markers with optimized specificity can be used at the stage of choosing diagnostic tactics for patients whose total PSA value is in the gray zone (between 4 and 10 ng/mL).

## 4. Discussion

Neoplastic transformation induces specific alterations in the cellular miRNA profile. These alterations are supposed to be translated into the composition of miRNAs released from cells into the extracellular space within SEVs. However, the total SEV population is extremely heterogeneous; so, small amounts of tissue-specific vesicles do not significantly affect the overall profile of circulating vesicles. Thus, for diagnostics, it is more considerable to use specific fractions of microvesicles. To create a diagnostic method, SEVs secreted by cells of certain tissue must be separated to detect cancer-indicative alterations in the miRNA content of these vesicles. First, we performed a proof-of-principle study by analyzing the miRNAs in SEVs bearing thyroid peroxidase and revealed the potent ability of this test to discriminate follicular adenoma and follicular cancer of the thyroid gland [15]. For prostate cancer, the effectiveness of the analysis of specific fractions of SEVs has already been highlighted in the work of Chiara Foroni et al. [30]. However, in this study, the vesicles with undisclosed cancer-specific surface marker were isolated by immune-capturing and multiple PC-relevant alterations of the androgen receptor were profiled in SEV-associated DNA. In our study, we explored other tactics and a different strategy. We aimed to isolated prostate-derived SEVs instead of cancer-specific vesicles; and we explored aptamer-based technology instead of antibody-based immune-capturing. We supposed that PC-associated alterations of miRNAs in prostate-derived SEV will have diagnostic potency. The obtained results confirmed our hypothesis; however, certain issues requiring further research were highlighted.

The first unexpected result was the high relative amount of PSMA(+)SEVs in plasma. The dot blot analysis cannot distinguish the signal from a few vesicles enriched by the marker of interest from a high number of vesicles with a low amount of the marker. Comparative analysis of certain markers’ expression in vesicles separated by stochastic optical reconstruction microscopy (STORM) would help resolve this issue and will be applied in the future. We suppose that PSMA is expressed at a similar or lower level than CD63 in prostate-derived SEVs, and the obtained ratio of PSMA to CD63 signal intensity reflects the ratio of PSMA(+) to CD63(+) vesicles. This interpretation of the results allows us to state that PSMA(+)SEVs constitute 20–24% of the total vesicular population. If this is true, PSMA(+)SEVs cannot be secreted exclusively by prostate cells. Thus, the fraction of PSMA(+)SEVs is not prostate-specific but rather enriched by prostate-derived SEVs. This conclusion justifies further optimization of the isolating technology using other prostate-specific markers and their combination.

The second interesting result of our investigation is the higher efficacy of complexes functionalized by DNA aptamers compared to complexes functionalized by antibodies. To explain this difference, we assumed similar affinity of both ligands to PSMA and calculated the number of ligand molecules apparently attached to the same amount of SPMBs. Thus, SPMBs were functionalized with PSMA–Apt in 100 μL of reaction mix containing 1.7 × 10^−6^ g of DNA aptamers with a molecular mass of 17 kDa, which corresponds to 60.2 × 10^12^ molecules per reaction. Likewise, SPMBs were functionalized with PSMA-Ab in a 100 μL of reaction mix containing 1.7 × 10^−6^ g of antibodies with a molecular mass of 150 kDa, which corresponds to 6.8 × 10^12^ molecules per reaction. This calculation indicates an eight-times higher density of SPMA binding sites on the surface of SPMB–PSMA–Apt complexes than on the surface of SPMB–PSMA-Ab complexes. Excluding the possible differences in the efficiency of the binding of ligands to SPMB and the affinity of interaction of ligands with PSMA, this calculation can, in part, explain the higher PSMA(+)SEVs binding capacity of SPMB–PSMA–Apt complexes and may stimulate further development of aptamer-based technology. Also, to prove the effectiveness of using the SPMB-PSMA-Apt complex (via flow cytometry), for determining significant results, magnetic particles without PSMA-aptamer incubated with vesicles labeled with Vibrant-Dil were used as a limit of detection and negative control. It would be more correct to use the lowest concentration of PSMA(+) vesicles that can be detected by a developed method as a limit of detection. Unfortunately, there is no method available to determining this concentration. Therefore, we are planning to continue working on the development of a method to estimate the concentration of PSMA (+) SEVs.

Finally, we want to indicate the most important issue that requires extensive further investigation to advance the proposed technology from bench to bedside. In our study, we empirically selected four PC-related miRNAs and revealed a method to improve their diagnostic potency. We considered that the cancer-related expression alterations of these molecules translate to the miRNA content of prostate-derived SEVs. However, it was reported that the packaging of miRNAs into exosomes or other vesicles is an active and regulated process, and the cellular miRNA pattern is not the same as the vesicular miRNA pattern. This indicates the importance of deeper analysis and miRNA profiling of prostate-derived SEVs isolated from the plasma of healthy donors and PC patients. The selection of highly relevant miRNA markers would increase the overall diagnostic potency of the proposed method.

## Figures and Tables

**Table 1:** Parameters of diagnostic significance for the selected miRNAs.

Statistic	Mir-145	Mir-221	Mir-451a	Mir-141
Sensitivity	65.45	70.71	66.67	74.55
Specificity	83.33	70.00	61.29	60.00
Positive Predictive Value	87.80	81.25	75.00	77.36
Negative Predictive Value	56.82	56.76	51.35	56.25
Accuracy	71.76	70.59	64.71	69.41

## Data Availability

Data are available upon request.
